# Metabolic clusters of breast cancer in relation to gene- and protein expression subtypes

**DOI:** 10.1186/s40170-016-0152-x

**Published:** 2016-06-27

**Authors:** Tonje H. Haukaas, Leslie R. Euceda, Guro F. Giskeødegård, Santosh Lamichhane, Marit Krohn, Sandra Jernström, Miriam R. Aure, Ole C. Lingjærde, Ellen Schlichting, Øystein Garred, Eldri U. Due, Gordon B. Mills, Kristine K. Sahlberg, Anne-Lise Børresen-Dale, Tone F. Bathen, The Oslo Breast Cancer Consortium (OSBREAC)

**Affiliations:** Department of Circulation and Medical Imaging, Norwegian University of Science and Technology (NTNU), Trondheim, Norway; K.G. Jebsen Center for Breast Cancer Research, Institute of Clinical Medicine, Faculty of Medicine, University of Oslo, Oslo, Norway; St. Olavs Hospital, Trondheim University Hospital, Trondheim, Norway; Department of Food Science, Faculty of Science and Technology, Aarhus University, Årslev, Denmark; Department of Cancer Genetics, Institute for Cancer Research Oslo University Hospital, The Norwegian Radium Hospital, Oslo, Norway; Department of Computer Science, University of Oslo, Oslo, Norway; Centre for Cancer Biomedicine, University of Oslo, Oslo, Norway; Section for Breast and Endocrine Surgery, Oslo University Hospital, Ullevål, Oslo Norway; Department of Pathology, Oslo University Hospital, Oslo, Norway; Department of Systems Biology, The University of Texas M.D. Anderson Cancer Center, Houston, TX USA; Department of Research, Vestre Viken, Drammen, Norway

**Keywords:** Metabolomics, HR MAS MRS, Breast cancer subgroups, Metabolic cluster, Extracellular matrix

## Abstract

**Background:**

The heterogeneous biology of breast cancer leads to high diversity in prognosis and response to treatment, even for patients with similar clinical diagnosis, histology, and stage of disease. Identifying mechanisms contributing to this heterogeneity may reveal new cancer targets or clinically relevant subgroups for treatment stratification. In this study, we have merged metabolite, protein, and gene expression data from breast cancer patients to examine the heterogeneity at a molecular level.

**Methods:**

The study included primary tumor samples from 228 non-treated breast cancer patients. High-resolution magic-angle spinning magnetic resonance spectroscopy (HR MAS MRS) was performed to extract the tumors metabolic profiles further used for hierarchical cluster analysis resulting in three significantly different metabolic clusters (Mc1, Mc2, and Mc3). The clusters were further combined with gene and protein expression data.

**Results:**

Our result revealed distinct differences in the metabolic profile of the three metabolic clusters. Among the most interesting differences, Mc1 had the highest levels of glycerophosphocholine (GPC) and phosphocholine (PCho), Mc2 had the highest levels of glucose, and Mc3 had the highest levels of lactate and alanine. Integrated pathway analysis of metabolite and gene expression data uncovered differences in glycolysis/gluconeogenesis and glycerophospholipid metabolism between the clusters. All three clusters had significant differences in the distribution of protein subtypes classified by the expression of breast cancer-related proteins. Genes related to collagens and extracellular matrix were downregulated in Mc1 and consequently upregulated in Mc2 and Mc3, underpinning the differences in protein subtypes within the metabolic clusters. Genetic subtypes were evenly distributed among the three metabolic clusters and could therefore contribute to additional explanation of breast cancer heterogeneity.

**Conclusions:**

Three naturally occurring metabolic clusters of breast cancer were detected among primary tumors from non-treated breast cancer patients. The clusters expressed differences in breast cancer-related protein as well as genes related to extracellular matrix and metabolic pathways known to be aberrant in cancer. Analyses of metabolic activity combined with gene and protein expression provide new information about the heterogeneity of breast tumors and, importantly, the metabolic differences infer that the clusters may be susceptible to different metabolically targeted drugs.

**Electronic supplementary material:**

The online version of this article (doi:10.1186/s40170-016-0152-x) contains supplementary material, which is available to authorized users.

## Background

Breast cancer accounts for 25 % of newly diagnosed cancers and 15 % of cancer deaths among women worldwide [1]. It is a heterogeneous disease [2] with high diversity in prognosis and response to treatment. Identification of underlying mechanisms contributing to this heterogeneity may reveal new cancer targets and clinically relevant subgroups and has thus been the focus of many recent studies [3–5].

Searching for genetic features causing the variation in breast cancers, Perou et al. used gene expression analyses followed by hierarchical clustering and defined naturally occurring molecular subtypes [4, 6]. These subtypes are named basal-like, luminal A, luminal B, Erb-B2+ (Her2 enriched), and normal-like, and are found to be associated with tumor characteristics and clinical outcome; patients with basal-like tumors having the shortest and luminal A the longest relapse-free survival [6]. A centroid-based method called prediction analysis of microarrays 50 (PAM50), which uses the expression of 50 genes to classify breast cancer into these five intrinsic subtypes was later established and is now broadly implemented [7].

Proteins are the ultimate cellular effectors of pathways and networks within cells, tissues, and organisms. Although protein levels are dependent on mRNA expression, not all mRNA will be translated into protein and further protein levels are also influenced by protein stability. In a study by Myhre et al. only 22 of 52 quantified breast cancer-related proteins were found to correlate with mRNA expression levels [8] and similar low levels of correlation have been seen in large scale studies [9, 10]. Protein expression subtypes of breast cancer could give further understanding of underlying mechanisms causing heterogeneity [11]. Based on the expression of 171 breast cancer-associated proteins detected by reverse phase protein array (RPPA), six breast cancer subtypes, called RPPA subtypes, have been defined [5]. Four of these subgroups were in high accordance with the gene expression profiles of the PAM50 subtypes and named accordingly; Basal, Her2, luminal A, and luminal A/B. In addition, two new subgroups were defined; reactive I and reactive II, based on expression of proteins possibly produced by the surrounding microenvironment.

The chemical processes controlled by proteins involve metabolites as intermediates or end-products. In metabolomics, metabolite levels are measured to gather the final downstream information of ongoing cellular processes. Which processes are active at a specific time point is strongly influenced by environmental factors like diet and drugs as well as disease state. Well-established metabolic differences have been observed when comparing cancer cells to normal cells. Cancer cell energy production frequently depends on increased glycolysis and production of lactate from glucose regardless of access to oxygen, in contrast to normal cells which produce pyruvate and lactate in aerobic conditions [12]. Also, to produce macromolecules/biomass, mitochondrial metabolism is reprogrammed [13]. Altered metabolism has therefore been included as one of the emerging hallmarks of cancer [14]. In breast cancer, metabolic differences between cancer tissue and normal adjacent tissue have been studied by the magnetic resonance spectroscopy (MRS) method high-resolution magic-angle spinning (HRMAS) MRS [15]. Using this technique, metabolic profiles and biomarkers predicting long-term survival for locally advanced breast cancer [16], node involvement of patients with infiltrating ductal carcinoma [17], and 5-year survival for ER positive patients [18] have been identified.

Merging transcriptomics and metabolomics led to the discovery of three luminal A subgroups with distinct metabolic profiles and significant differences within gene set expression in a study by Borgan et al. [19]. The aim of the current study was to establish clusters of breast cancer based on the metabolic expression using an approach similar to Borgan et al., but in a larger cohort of patients including all PAM50 subgroups. This approach reveals the main metabolic differences between untreated breast tumors. In addition, the combination of the metabolic clusters with transcriptomics and protein expression data provide an opportunity for information gain from each -omics technology, giving further characterization of the defined metabolic clusters.

## Methods

### Patients and tissue samples

Primary breast carcinoma samples from 228 patients at the Oslo University Hospital (Radium Hospital and Ullevål Hospital) were collected in the time period 2006–2009 as part of the Oslo2 study. The samples were fresh frozen after surgery and stored at −80 °C. The tumors were divided into smaller pieces depending on their size, and one of them was selected for this study. The samples were cut into three sections where the edges of the two outer pieces were used for histological evaluation (including estrogen receptor (ER) status and tumor cell percentage), and an adequate part of the mid pieces were used for HR MAS MRS experiments to obtain metabolic profiles. The remnants of all three pieces were pooled and cut into smaller pieces with scalpel, depending on the size of the tumor and divided into fractions used for extraction of DNA, RNA, and protein. Due to high lipid content, HR MAS MRS was performed on a second piece from the same tumor for 13 of the samples. A total of 228 samples were analyzed by MR spectroscopy, of which 201 and 217 were analyzed for gene expression by arrays and protein expression using RPPA, respectively, leaving a total of 191 samples analyzed by all three methods. Patient and tumor characteristics are shown in Table 1.

**Table 1:** Patient and tumor characteristics

	Total	Mc1	Mc2	Mc3
Number of patients	228	58	58	112
Age (years), mean (range)	55.5 (31.8–81.1)	58.0 (33.2–80.8)	58.6 (40.9–81.1)	52.7 (31.8–73.9)
Clinical classification				
Histology				
Ductal	186	52	37	97
Lobular	21	4	11	6
Medullary	0	0	0	0
Ductal carsinoma in situ (DCIS)	4	0	4	0
Metaplastic	1	0	1	0
Mucinous	4	0	2	2
Tubular	4	1	1	2
Mixed	2	1	0	1
Papillary	0	0	0	0
NA	6	0	2	4
Primary tumor				
Tx or *NA*	9	1	3	5
T0	0	0	0	0
pTis	4	0	4	0
T1	113	31	28	54
T2	93	24	21	48
T3	9	2	2	5
T4	0	0	0	0
Grade				
I	31	8	10	13
II	93	20	24	49
III	97	30	21	46
NA	7	0	3	4
Node status				
N0	133	34	36	63
N1(mi)	8	3	3	2
N1	59	17	13	29
N2	14	2	3	9
N3	8	2	1	5
NA	6	0	2	4
Receptor status				
HER2+	26	7	7	12
HER2−	192	51	45	96
ER+	178	49	42	87
ER−	40	9	10	21
PR+	155	39	36	80
PR−	63	19	16	28
NA	10	0	6	4

*NA*, not available

### HR MAS MRS spectra

HR MAS MRS spectra were acquired from tissue samples (mean sample weight 7.3 mg ± 2.6 mg) on a Bruker Avance III 600 MHz/54 mm US (Bruker, Biospin GmbH, Germany) equipped with a 1H/13C MAS probe with gradient aligned with the magic angle (Bruker, Biospin GmbH, Germany). Spin-echo spectra were recorded using a Carr-Purcell-Meiboom-Gill (cpmg) pulse sequence (cpmgpr1d; Bruker). For experimental details and information about data processing, see Additional file 1.

Forty-three samples were excluded from the original sample cohort of 271 samples due to large lipid content. The spectral region between 1.40 and 4.70 ppm was chosen for further analysis excluding lipid peaks at 4.36–4.27, 2.88–2.70, 2.30–2.20, 2.09–1.93, and 1.67–1.50 ppm. After removal of the lipid residuals, the spectra were mean normalized by dividing each spectral variable to the average spectral intensity. This is done to account for differences in tumor cell percentage and sample weight, as it can be assumed that most of the lipid signals from breast samples do not originate from cancer cells.

### Protein experiments and protein expression subtyping

Protein levels were determined using reverse phase protein array (RPPA), a platform where single protein levels can be measured across a series of samples simultaneously [20]. One hundred fifty primary antibodies were used to detect breast cancer-related proteins (Additional file 2: Table S1). For analytical details, see Additional file 1.

The samples underwent consensus clustering with an option for four or five groups. The best fit on consensus clustering identified five groups, luminal, HER2, basal, and reactive I and II subsets as defined in The Cancer Genome Atlas Network data set [5].

### mRNA expression profiling and gene expression subtyping

Total RNA was isolated with TRIzol (Invitrogen, Carlsbad, CA, USA). Expression of mRNA was measured using SurePrint G3 Human GE 8x60K (Aglient Technologies) according to the manufactory’s protocol (one-color microarray-based gene expression analysis, low input Quick Amp Labeling, v.6.5, May 2010) and 100 ng RNA was used as input for labeling. Arrays were log2-transformed, quantile normalized, and hospital adjusted [21]. Values corresponding to probes with identical Entrez ID were averaged to form a single expression value per gene.

The PAM50 subtype algorithm [7] was used to assign a subtype label to each sample as previously described [22].

### Statistical analysis

#### Subgrouping with cluster analysis of metabolic data

Hierarchical cluster analysis (HCA) was performed with Euclidean distance as the distance parameter and Ward’s method (furthest inner square distance) as the clustering distance (Statistical toolbox, Matlab R2013b, The Mathworks, Inc., USA) on the preprocessed metabolic spectra. Similar spectra based on the distance measures cluster together. The dendrogram was cut to give three clusters. To evaluate the robustness of the three HCA clusters, partial least square discriminant analysis (PLS-DA) model, using the cluster group for classification was carried out and classification accuracy was evaluated. For details, see Additional file 1.

#### Analysis of metabolic profiles

Metabolite assignments were performed based on literature values [23]. Relative metabolite quantification was performed by peak integration of fixed regions corresponding to the metabolite of interest. In total, the level of 18 metabolites were calculated. Kruskal-Wallis test was performed to compare metabolite levels between clusters. Calculated *p* values were corrected for multiple testing by The Benjamini Hochberg false discovery rate (FDR) in Matlab, and the differences were considered statistically significant for adjusted *p* ≤ 0.05.

#### Analysis of subtype and clinical distributions

Differences in the distributions of RPPA and PAM50 subtype as well as that of other clinical characteristics of the tumors between the different metabolic clusters were tested for significance using Fisher’s exact test for count data (R 2.15.2). Calculated *p* values were corrected for multiple testing by The Benjamini Hochberg FDR, and the differences were considered statistically significant for adjusted *p* < 0.05.

#### Analysis of gene expression data

Significance analysis of microarrays (SAM) was used to identify differentially expressed gene between the metabolic clusters [24]. SAM analysis was performed using 21851 genes from 42405 mRNA probes. The expression analysis was performed in R 2.15.2 [25] with the cluster group as the dependent variable and a total of 100 permutations. *T* statistics/Wilcoxon statistics were calculated using multiclass comparisons and two-class unpaired tests while comparing two clusters. The differences were considered statistically significant for adjusted *p* < 0.01.

DAVID, an online network analysis tool [26], was used to search for biological functions within gene sets. DAVID was performed on the gene list over for each of the class comparisons produced by SAM. Official gene symbol was selected as gene identifier. The functional annotation clustering report of this software reports similar annotations together, where the member of a cluster have similar biological meaning due to sharing of similar gene members.

Gene set enrichment analysis (GSEA) was used to identifying sets of genes that were enriched in the metabolic clusters [27, 28]. During each cluster comparison, genes were ranked depending on calculated absolute signal-to-noise ratio (Eq. 1), where *μ* and *σ* are the mean and standard deviation, respectively.

1 $$ \mathrm{abs}\left(\frac{\mu_A-{\mu}_B}{\sigma_A+{\sigma}_B}\right) $$

High absolute signal-to-noise ratio will represent genes that are more likely to be “class markers” in the comparison because of high difference in expression.

The gene set C5 (gene ontology (GO) gene sets) available from the Molecular Signatures Database (MSigDB) [29] from The Broad Institute was chosen for evaluation of enrichment. One thousand four (of 1454) gene sets from this data base passed the filtering of lacking any gene from the expression data followed by minimum and maximum size of 15 and 500 genes, respectively. For each comparison, 1000 permutations on phenotypes were performed and FDR cutoff was set to 25 % (recommended in the manual).

#### Integrated pathway analysis

To combine transcriptomics and metabolic data the “Integrated pathway analysis” tool in MetaboAnalyst 3.0 software was used [30]. Genes with adjusted *p* < 0.05 from SAM analysis and metabolites differently expressed between the clusters were used as input. Pathways with *p* values ≤0.05 were interpreted as significant.

## Results

### Three main metabolic clusters of breast cancer

**Fig. 1:**
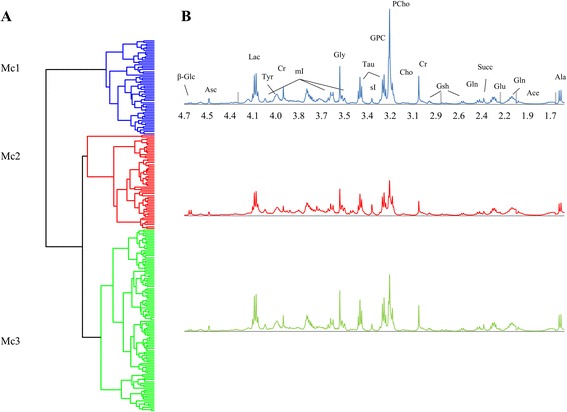
Metabolic subtyping of breast cancer tissue samples using HCA. **a** The HR MAS MRS spectra for 228 samples was clustered using Euclidean distance and Wards linkage as similarity measure which separated the samples into three metabolic clusters (Mc); Mc1, Mc2, and Mc3. **b** Mean spectra for the three metabolic clusters. *β-Glc* β-glucose, *Asc* ascorbate, *Lac* lactate, *Tyr* tyrosine, *Cr* creatine, *mI* myoinositol, *Gly* glycine, *Tau* taurine, *sI* scylloinositol, *GPC* glycerophosphocholine, *PCho* phosphocholine, *Cho* choline, *Gsh* glutathione, *Gln* glutamine, *Succ* succinate, *Glu* glutamate, *Ace* acetate, *Ala* alanine. *Grey bars* indicate removed spectral regions (containing lipid peaks)

From the spectral data of 228 breast tumors, hierarchical clustering gave a dendrogram divided in three metabolic clusters (Mc) (Fig. 1a) Mc1, Mc2, and Mc3. The mean spectra of the clusters are illustrated in Fig. 1b.

**Fig. 2:**
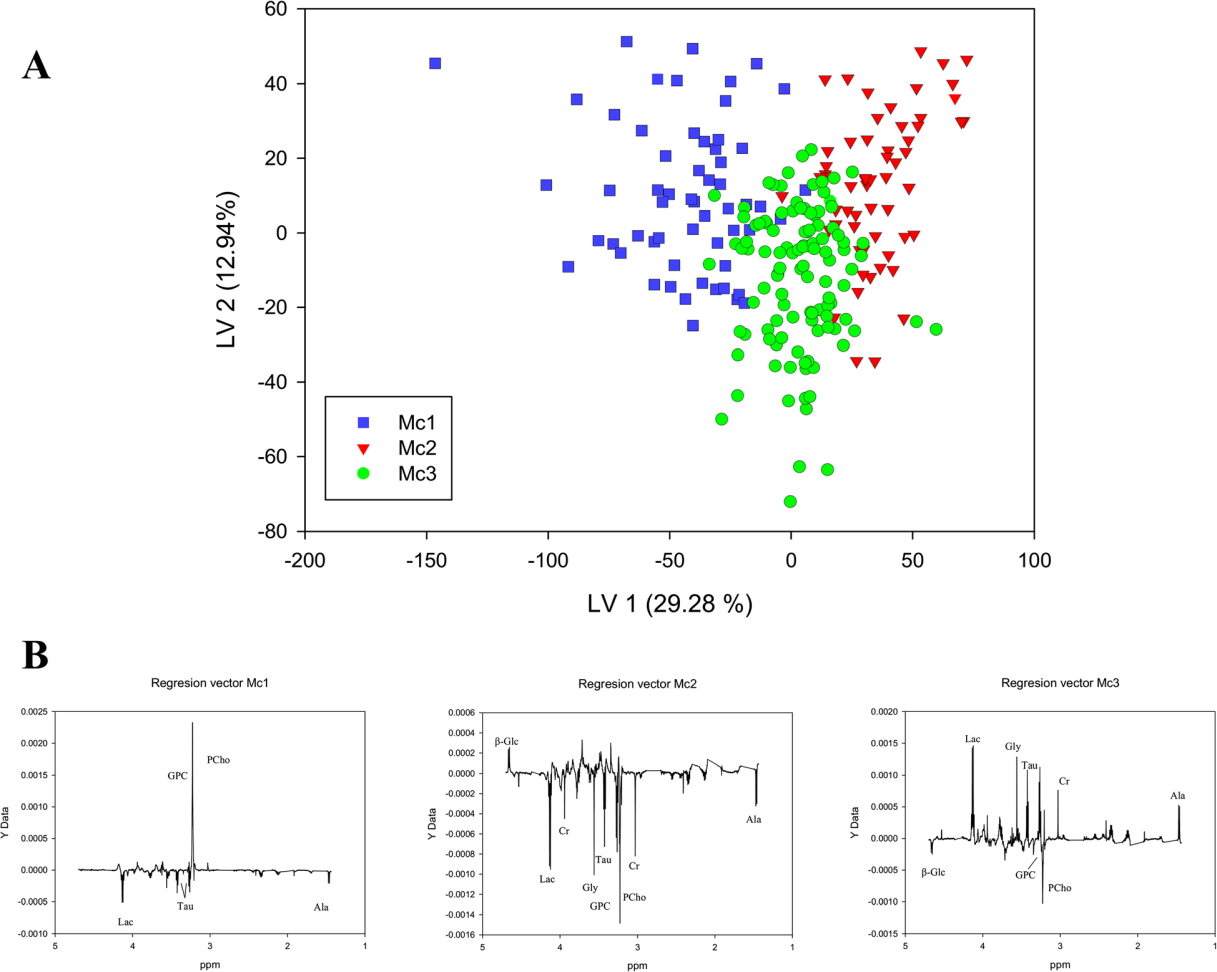
Results from PLS-DA of metabolic clusters. **a** Score plot of the two first latent variables explaining 42.2 % of the *X*-variance and 28.2 % of the *Y*-variance. (**b**) Regression vectors for the three metabolic clusters (Mc)

**Fig. 3:**
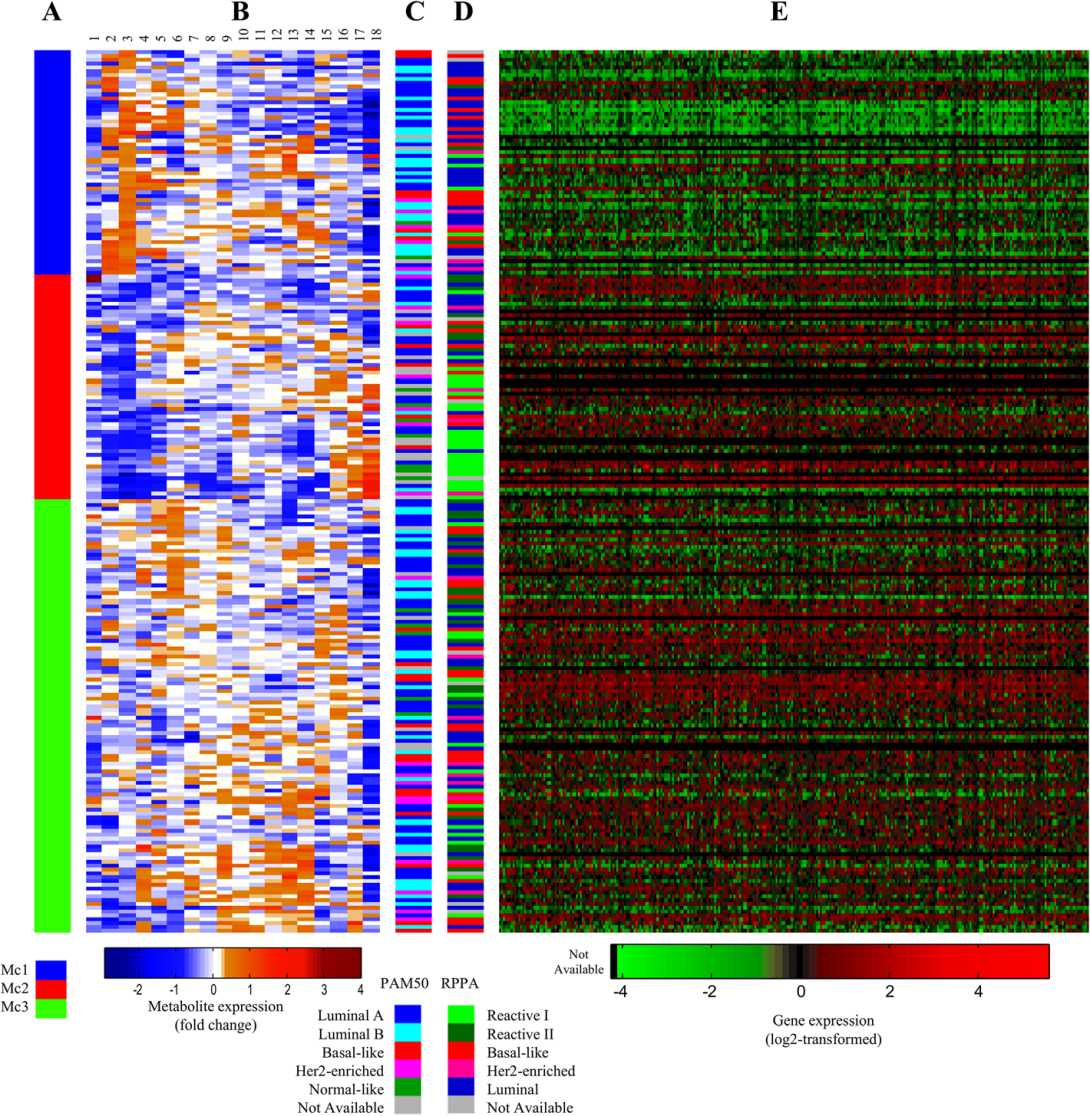
Main differences between metabolic subtypes. **a** Metabolic cluster label from HCA with Euclidean distance and Wards linkage of HR MAS MR spectra of samples. The samples clustered in three groups called Mc1, Mc2, and Mc3. **b** Fold change in expression levels of (1) scylloinositol, (2) GPC, (3) PCho, (4) creatine, (5) ascorbate, (6) taurine, (7) GSH, (8) tyrosine, (9) lactate, (10) glutamate, (11) succinate, (12) glutamine, (13) glycine, (14) alanine, (15) choline, (16) myoinositol, (17) acetate, and (18) glucose. *Blue regions* in the heat map represent decreased levels while *red levels* represent increased metabolite levels. **c** PAM50-subtypes. **d** RPPA-subtype. **e** Gene expression levels (quantile normalized, log2-transformed) for the 277 overlapping significant genes (SAM, adjusted *p* < 0.01) between Mc1 and Mc3. The genes have been clustered

The prediction of the metabolic clusters by PLS-DA resulted in a model with two valid latent variables LVs (Fig. 2a). The clusters Mc1 and Mc2 were well separated in the score plot of LV1 and LV2, while most Mc3 samples had low values of LV2. Classification accuracy was found to be 91.1, 88.7, and 69.9 %, respectively, for the three clusters. Permutation testing showed that all three clusters had significantly different metabolic profiles (*p* < 0.001). The regression vectors for each of the clusters (Fig. 2b) indicate each metabolite’s influence on the cluster prediction. The regression vector for Mc1 showed that high levels of glycerophosphocholine (GPC) and phosphocholine (PCho) and low levels of lactate (Lac), taurine (Tau), and alanine (Ala) were important for the class prediction result. For Mc2, high levels of β-glucose (β-Glc) were important as well as low levels of Lac, creatine (Cr), glycine (Gly), Tau, GPC, PCho, and Ala. Mc3 had a regression profile with low β-Glc, GPC, and PCho levels, and high Lac, Gly, Tau, Cr, and Ala levels. Univariate comparison of metabolite levels (Additional file 2: Table S2) between the three clusters revealed that 15 out of 18 metabolites analyzed were found to be significantly different (adjusted *p* < 0.05) between at least two of the clusters (Table 2). A combination of metabolic cluster labels and heatmap of metabolite fold change further illustrate this (Fig. 3).

**Table 2:** Metabolite levels for the metabolic clusters

	Mc1 (*n* = 58)		Mc2 (*n* = 58)		Mc3 (*n* = 112)		Adjusted *p* value	Significant between
Metabolite name	Mean	SE	Mean	SE	Mean	SE		
Beta-d-glucose	30.0	27.7	71.7	55.2	32.3	16.3	3.62E-09	Mc2 vs rest
Ascorbate	40.0	17.1	28.8	8.6	38.3	13.6	1.02E-05	Mc2 vs rest
Lactate	259.5	73.0	229.4	57.6	303.6	76.7	4.98E-09	Mc3 vs rest
L-tyrosine	407.5	56.5	352.8	82.6	405.9	62.4	1.22E-04	Mc2 vs rest
Glycine	187.0	80.8	152.3	41.9	195.7	68.8	1.04E-04	Mc2 vs Mc3
Myoinositol	163.7	47.0	217.7	53.5	196.1	54.3	9.44E-07	all
Taurine	332.2	122.7	330.2	84.0	369.3	99.3	0.017	Mc1 vs Mc3
Scylloinositol	55.0	16.2	94.7	186.5	62.5	32.1	0.138	NS
Glycerophosphocholine	210.0	91.6	107.9	33.6	151.2	48.4	4.44E-12	all
Phosphocholine	552.0	131.1	216.8	66.8	327.2	69.9	9.59E-33	all
Choline	135.2	44.6	120.3	37.7	132.9	42.2	0.128	NS
Creatine	149.9	64.2	93.2	33.7	136.0	52.1	1.41E-09	Mc2 vs rest
Glutathione	57.5	13.8	50.9	13.9	58.1	14.5	0.011	Mc2 vs Mc3
Glutamine	134.4	41.3	134.3	30.2	145.4	43.6	0.223	NS
Succinate	58.0	15.7	53.6	10.6	62.2	15.7	0.003	Mc2 vs Mc3
Glutamate	237.9	61.3	266.2	63.3	277.5	61.2	1.95E-04	Mc1 vs rest
Acetate	32.7	9.0	48.4	17.2	40.3	13.1	7.89E-08	all
Alanine	82.6	36.6	66.0	24.9	95.1	33.8	6.56E-07	all

The values are calculated by integrated peak areas from normalized spectra to equal total areas. Kruskal-Wallis test was performed to compare metabolite levels between clusters, and *p* values were adjusted for multiple testing by The Benjamini Hochberg false discovery rate

*NS* not significant (adjusted *p* > 0.05)

Clinical parameters (tumor size, histology, grade, node status, hormone receptor status) were analyzed for differences in distribution among the metabolic clusters. Only histology was found to be significantly different between the clusters (adjusted *p* = 0.0144), where 11 of 21 lobular tumors and all ductal carcinoma in situ (DCIS) (*n* = 4) were classified as Mc2 (Table 1).

### Protein expression subtype (RPPA) distribution differs between the three metabolic clusters

The metabolic clusters were investigated for differences in distribution of PAM50 and RPPA subtypes. While PAM50 subtypes did not show increased frequency of occurrence in any of the metabolic clusters, (Fig. 3c, adjusted *p* = 0.138), RPPA distribution was significantly different (Fig. 3d, adjusted *p* = 1.43E-04) with only 9 % of the RPPA reactive I and II samples being classified as Mc1, and 44 % of Mc2 samples subtyped as reactive I. The complete distribution of PAM50 and RPPA subtypes is listed in Table 3.

**Table 3:** Distribution of PAM50 and RPPA subtype among the metabolic clusters

		Metabolic cluster		
	Total	Mc1	Mc2	Mc3
PAM50 subtype				
Luminal A	85	19 (35)	18 (43)	48 (46)
Luminal B	56	23 (42)	5 (12)	28 (27)
Basal	24	6 (11)	5 (12)	13 (13)
Her2 enriched	22	5 (9)	7 (17)	10 (10)
Normal-like	14	2 (4)	7 (17)	5 (5)
NA	27	4	15	8
Total	201	55	42	104
RPPA subtype				
Reactive I	43	4 (7)	24 (44)	15 (14)
Reactive II	36	3 (5)	8 (15)	25 (23)
Basal	47	16 (29)	8 (15)	23 (21)
Her2	18	5 (9)	4 (7)	9 (8)
Luminal	73	27 (49)	11 (20)	35 (33)
NA	11	3	3	5
Total	217	55	55	107

Values in brackets are each subtype’s percentage distribution within the metabolic clusters

*NA* not available

### SAM reveals only one metabolic cluster to have differences in gene expression

SAM was performed to identify expression differences between the metabolic clusters. Of the 21,851 genes, multiclass SAM showed that 696 were differently expressed between the metabolic clusters with adjusted *p* < 0.01 (Fig. 3e, Additional file 2: Table S3). Further investigation through two-class SAM revealed that Mc2 and Mc3 did not have significant differences in mRNA expression, while they had 413 and 617 genes upregulated, respectively, compared to Mc1 (Additional file 2: Tables S4 and S5, respectively). Out of these, 277 genes were found in both comparisons and upregulated compared to Mc1. DAVID software was used to investigate the biological interactions between genes that were found to be significantly differentially expressed between the metabolic clusters.

A total of 404 of the 413 significant genes from SAM between Mc1 and Mc2 were identified by DAVID. Functional Annotation Clustering resulted in 117 clusters (Top 10 in Additional file 2: Table S6), where the clusters with the highest enrichment scores were linked to signaling, extracellular region, and cell adhesion.

A total of 653 of the 671 significant genes from SAM between Mc1 and Mc3 were identified by DAVID. Functional Annotation Clustering resulted in 236 clusters (Top 10 in Additional file 2: Table S7), where the clusters with the highest enrichment scores were linked to extracellular matrix (ECM), cell adhesion, and basement membrane.

### Enrichment analysis shows gene expression differences to be related to ECM activity

Since Mc1 was found to have a gene expression pattern different from both Mc2 and Mc3 and these two clusters lacked statistically significant gene expression differences, Mc1 was compared to Mc2 and Mc3 combined in GSEA. This resulted in 146 of the gene ontology gene sets altered in Mc1 compared to Mc2 and Mc3 (Additional file 2: Table S8). Gene sets with the highest significance were classified with functions within collagen, ECM, and integrin binding. None of the gene ontology sets were significantly different when comparing Mc2 to Mc1 combined with Mc3, but 44 gene sets were significantly enriched when comparing Mc2 to Mc1 alone, with gene ontology terms relevant to ECM dominating the result (Additional file 2: Table S9). Eleven gene sets were significantly altered between Mc3 and Mc1 combined with Mc2 (Additional file 2: Table S10) and also here ECM-related findings were reported. One hundred fourteen gene sets were significantly different between Mc1 and Mc3, while none were significant between Mc2 and Mc3 (results not shown).

### Joint analysis of gene and metabolite expression shows differences in metabolic pathways

**Fig. 4:**
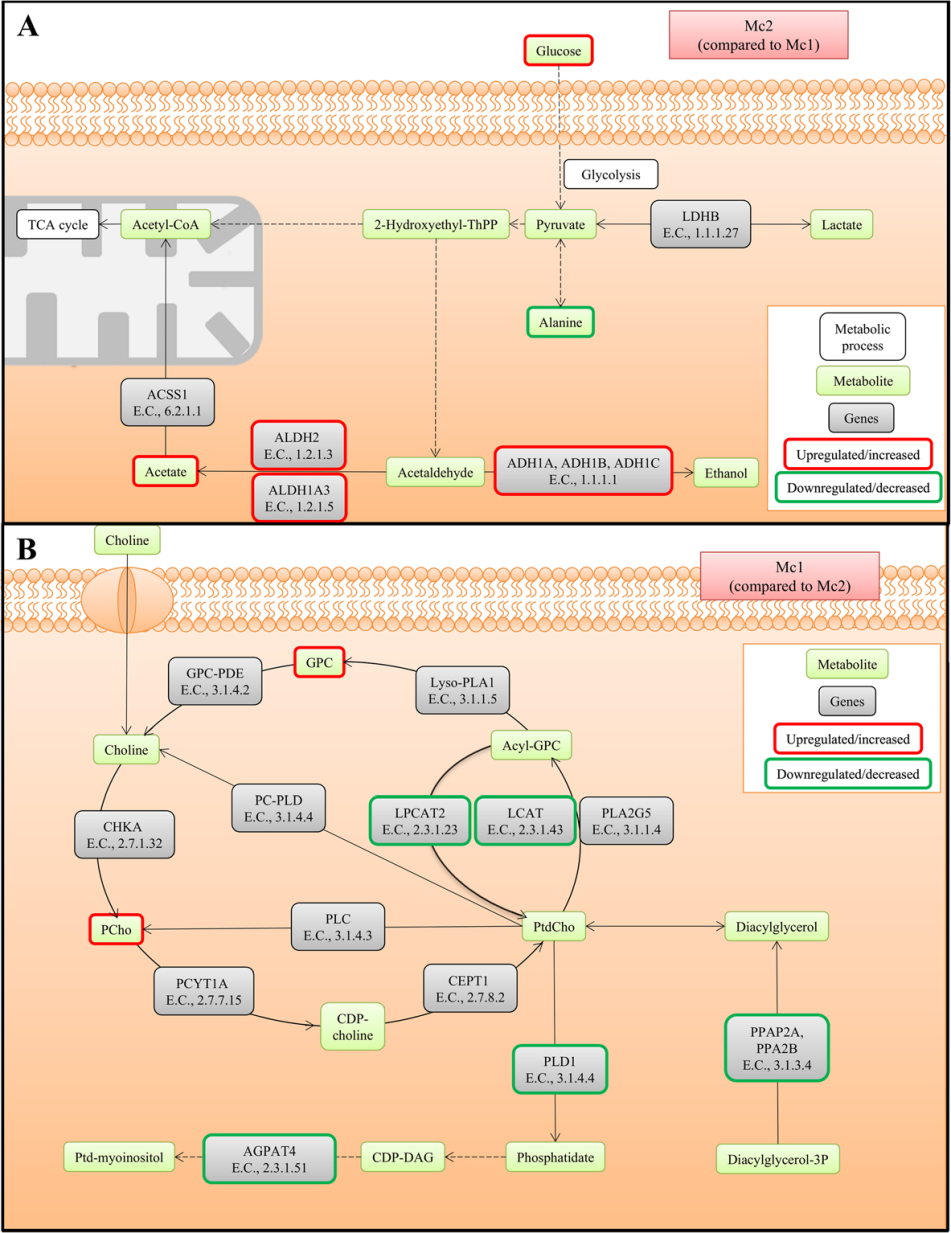
Illustration of metabolic pathways reported to have altered gene and metabolite expression by integrated pathway analysis (MetaboAnalyst). **a** Result within “glycolysis/glutaminolysis” genes and metabolites differently expressed in metabolic cluster (Mc) Mc2 compared to Mc1. Adapted from KEGG ID: hsa00010. *LDHB* lactate dehydrogenase B; *ADH1A/ADH1B/ADH1C* alcohol dehydrogenase 1A/1B/1C; *ALDH1A3*: aldehyde dehydrogenase 1 family member A3; *ALDH2* aldehyde dehydrogenase 2 family; *ACSS1* acetate CoA ligase; *TCA cycle* trucarboxylic acid cycle. **b** Result within “glycerophospholipid metabolism” of genes and metabolites differently expressed in Mc1 compared to Mc2. Adapted from KEGG ID: hsa00564. *CHKA* choline kinase alpha; *PCYT1A* phosphate cytidylytransferase 1; *CEPT1* choline/ethanolamine phosphotransferase 1; *PLA2G5* phospholipase A2; *LCAT* lecithin-cholesterol acyltransferase; *LPCAT2* lysophosphatidyl-choline acyltransferase; *PC-PLD* phospholipase D; *Lyso-PLA1* lysophospholipase I; *GPC-PDE* glycerophos-phocholine phosphodiesterase; *PLC* phospholipase C; *PLD1* phospholipidase D1; *PPAP2A, PPAP2B* phosphatidate phosphatase LPIN; *AGPAT4* 1-acylglycerol-3-phosphate O-acyltransferase

Integrated pathway analysis resulted in 12 significantly different metabolic pathways (*p* value <0.05) between Mc1 and Mc2 (Additional file 2: Table S11). The most significant pathway was “tyrosine metabolism’ with eight hits of genes and metabolites, but also “d-glutamine and d-glutamate metabolism,” “glycolysis/gluconeogenesis” (Fig. 4a), and “glycerophospholipid metabolism” (Fig. 4b) were among the significant pathways. Integrated pathway analysis resulted in four significantly different metabolic pathways (*p* value <0.05) between Mc1 and Mc3 (Additional file 2: Table S12). The most significant pathway was glycerophospholipid metabolism with nine hits, succeeded by d-glutamine and d-glutamate metabolism.

## Discussion

In the present work, metabolite, protein, and gene expression data from 228 breast tumors were combined to search for new insight into the heterogeneity of breast cancer. MR metabolite data was used to derive naturally occurring metabolic clusters, which were further combined with data from the proteomics and transcriptomics levels. We identified three significantly different metabolic clusters, Mc1, Mc2, and Mc3, with significant differences in gene expression and protein expression profiles, but not within PAM50 subgroups. The metabolic clusters could therefore contribute with additional information beyond the intrinsic gene sets for understanding breast cancer heterogeneity.

Of the three metabolic clusters, Mc1 was on a separate branch in the dendrogram indicating that the metabolic profile of this cluster was the most different. This cluster is defined by significantly higher levels of GPC and PCho, two choline-containing metabolites involved in the synthesis and degradation of phosphatidylcholine (PtdCho), a major component of cell membranes [31]. Altered choline metabolism has been considered an emerging hallmark for malignant transformations and has been detected in several cancer types including breast cancer [32]. PCho in particular has been suggested a biomarker of breast cancer [33]. Both GPC and PCho are confirmed elevated in tumor tissue compared to adjacent non-involved tissue from breast cancer patients [17], and a higher GPC/PCho-ratio has been reported in ER negative tumors [34, 35]. The latter was also observed for our cohort (results not shown); however, there was no significant difference in ER status between the three metabolic clusters. Thus, the high level of GPC and PCho is not resulting from differences in the distribution of estrogen receptor (ER) status. Interestingly, integrated pathway analysis showed that glycerophospholipid metabolism was the most significant pathway, when comparing Mc1 to Mc2. This metabolic pathway had eight hits including the metabolites GPC and PCho and genes *LCAT*, *LPCAT2*, *PPAP2A*, *PPAP2B*, *PLD1*, and *AGPAT4*. Downregulation of the expression of these genes in Mc1 indicate a less active degradation of PtdCho causing an accumulation of GPC and PCho, thus explaining the higher levels of GPC and PCho in Mc1. Furthermore, *LPCAT2* is involved in the reaction where the GPC precursor (acyl-GPC) is converted into PtdCho. Lower expression of this gene may explain why the GPC precursor is directed to the production of GPC instead of PtdCho. The same hits were obtained when Mc1 was compared to Mc3. In addition, *PLA2G5*, one of the enzymes degrading PtdCho to acyl-GPC, is downregulated in Mc1 compared to Mc3, further supporting that Mc1 has an altered PtdCho metabolism.

The levels of PCho and GPC were higher in Mc1 compared to the two other clusters, but no significant difference in the expression of choline kinase alpha (*CHKA)* could be detected in the SAM analysis. However, univariate analysis confirmed that *CHKA* expression was significantly higher in Mc1. This is in agreement with previous findings revealing a positive correlation between levels of PCho and GPC and expression of *CHKA* [34, 36]*.*

For Mc1 compared to Mc2 through integrated pathway analysis, d-glutamine and d-glutamate metabolism has only two hits, but comes out as significant because of the small number of genes and metabolites within this pathway. Interestingly, the gene *GLS* which catalyzes the conversion of glutamine to glutamate is downregulated in Mc1, the cluster with lowest levels of glutamate. Glutamine metabolism is considered a therapeutic target as some cancer cells exhibit high uptake and addiction to this nonessential amino acid [37]. Since there were no differences in glutamine levels of Mc1 and Mc2, less glutamate in Mc1 could indicate that more glutamine is directed towards other metabolic pathways necessary for proliferation, glutathione needed for reducing power or further that glutamate is rapidly metabolized in cells through the TCA cycle or other mechanisms.

The distribution of protein subtypes (RPPA) was significantly different between the metabolic clusters, whereas no significant differences in the distribution of PAM50 subtypes were found. Thus, the metabolic difference between Mc1, Mc2, and Mc3 is not a result of intrinsic subtypes and might therefore contain additional information for understanding breast cancer heterogeneity. Among the tumors clustered in Mc1, 12 % were classified as RPPA-reactive (either I or II) while 49 % were classified as RPPA-luminal. The reactive RPPA subtypes have a characteristic protein expression pattern probably produced by the microenvironment [5], indicating less microenvironmental activity within Mc1. Mc1 also had downregulation of several genes involved in processes within the ECM of the stroma compared to both Mc2 and Mc3. As ECM changes can drive cancer behavior [38], these genetic differences between Mc1 and Mc2 might be of prognostic relevance. In fact, differences in expression of ECM-related genes have been used to stratify breast carcinomas into four groups, where the subgroup ECM1 have the worst prognosis [39]. ECM classification was not performed on this cohort. However, 34 of 43 genes that clustered with a tendency of being downregulated in ECM1 and ECM2 were also found to be downregulated in Mc1. In addition, only 5 of 46 genes reported to be downregulated in ECM2 compared to ECM1 were downregulated in Mc1 (results extracted from SAM analyses, Additional file 2: Table S6–S7). These results support the contention that Mc1 tumors have an ECM signature similar to the reported ECM2 tumors. ECM2 did not show significant difference in disease outcome compared to ECM3 and ECM4, but had better prognosis than ECM1 tumors [39].

Mc2 has a metabolic profile with significant higher glucose level and at the same time lower levels of most of the other metabolites compared to one or both of the remaining clusters. High glucose level could reflect lower glucose consumption, inferring a lower demand for energy within these tumors. Glycolysis/gluconeogenesis came out as a significant pathway when Mc1 was compared to Mc2 during integrated pathway analysis with two metabolite hits and five gene hits. For the most significant metabolite, glucose, the levels are higher in Mc2 compared to Mc1. Glucose is the main source of energy for mammalian cells, either through aerobic glycolysis (production of lactate even in the presence of oxygen) or tricarboxylic acid (TCA) cycle and oxidative phosphorylation. For normal proliferating cells and cancer cells, which both have an increased energy demand, a glycolytic switch is often observed (higher glycolytic rate) [12]. The increased glycolysis is followed by fermentation of the pyruvate to lactate (Warburg effect), in contrast to the conversion of acetyl CoA through the TCA cycle that occurs in normal non-proliferating cells. Increased glucose consumption is commonly used in tumor detection by using a glucose analogue and positron emission tomography (PET) [40] and has shown to correlate with poor prognosis and tumor aggressiveness [12]. However, not all breast cancers are detected by PET. Here, we expect lower sensitivity in detection of Mc2 tumors due to the possible difference in glycolytic rate. None of the genes with hits in glycolysis/gluconeogenesis for the comparison of Mc1 and Mc2 could directly explain the high glucose levels of Mc2 tumors, but altered expression of the genes indicates pyruvate being guided towards the TCA cycle rather than lactate production. Two of the alternative fates of pyruvate showed significantly higher levels (alanine) or levels approaching significance (lactate, adjusted *p* = 0.056), supporting a higher glycolytic rate in Mc1 and that the pyruvate produced is not directed to metabolism in the TCA cycle. The significantly lower acetate levels in Mc1 compared to Mc2 could be linked to *ALDH1A3* and *ALDH2* downregulation, since the enzymatic product of these genes catalyzes the reversible reaction where acetaldehyde is converted to acetate.

Both DAVID and GSEA showed that many of the genes found to be downregulated in Mc1 and consequently upregulated in Mc2 were related to ECM activity. Mc2 had the highest percentage of RPPA-reactive I with 44 % of Mc2 tumors classified as this protein subtype, also related to stromal changes. Together with the metabolic finding, this implies that Mc2 tumors have cancer cells with low proliferating rate and at the same time ongoing changes within the ECM of the stroma. Mc2 tumors also had a higher frequency of lobular and ductal carcinoma in situ, indicating metabolic differences between histological subtypes of breast cancer which should be further investigated.

Mc3 has the highest lactate levels of all three clusters and higher glycine level than Mc2. These metabolites have been related to poor prognosis in ER positive patients [18], and higher levels of glycine is also associated with poor prognosis in a study irrespective of ER status [41]. Although the ER-positive patients are equally distributed among our reported metabolic clusters, Mc3 expressed higher levels of both of these metabolites compared to Mc2. Moestue et al. detected differences in the expression of genes involved in choline degradation that could explain higher glycine concentrations in the poor-prognosis basal-like breast cancer xenograft model compared to luminal-like [42]. Five of the genes described by Moestue et al. were significantly upregulated in Mc3 compared to Mc1; *AGPAT4*, *PPAP2B*, *PPAP2A*, *LCAT*, and *PLD1*. Of these, *LCAT* and *PLD1* are directly involved in choline metabolism. *LCAT* catalyze the conversion of PtdCho to acyl-GPC while *PLD1* catalyzes the conversion of PtdCho to choline. Higher GPC levels, but no difference in choline levels in Mc3 compared to Mc1 indicates that a higher amount of GPC is converted to choline in Mc3, and further contributing to higher glycine levels through choline degradation.

Mc3 shares similarities with a previously reported metabolic subgroup of luminal A tumors with significantly lower levels of glucose, higher levels of alanine, and nearly significantly higher lactate levels [19]. In Mc3, we also see a significant higher level of lactate. Since one of the main sources of alanine is pyruvate, which also is the source for lactate, it appears that Mc3 is a cluster with a switch in glycolytic activity.

The majority of Mc3 tumors were classified as RPPA-luminal, similar to Mc1. In contrast to Mc1, Mc3 had a higher percentage of RPPA–reactive II tumors, probably linked to changes in stromal content. Also, gene expression wise, this was observed by significantly different gene expressions linked to ECM activity and the gene expression profile of Mc3 was found similar to the previously reported ECM3 or ECM4 subtypes [39].

In this study, information flow between the transcriptomics, proteomics, and metabolomics levels is illustrated; at the transcriptomics level, only one of the metabolic clusters shows difference in gene expression compared to the two others, while at the proteomics level, there is difference between all three clusters. Combining these findings, Mc1 is expected to have the worst prognosis due to the distinct gene expression profile and the alterations in both glycerophospholipid metabolism and evidence of increased glycolytic rate. However, this has to be validated when 5-year follow-up of this cohort is available. The main metabolic characteristics, especially of Mc1 and Mc3, have been proposed as treatment targets that could improve the therapeutic effect [43]. Cancer therapy targeting *CHKA*, the enzyme responsible for PCho production from choline, causes tumor growth arrest and apoptosis in preclinical models [44], while treatment targeting glycolytic enzymes in combination with chemotherapy has been shown to re-sensitize cancer cells that had become resistant to treatment [43]. Metabolic classification as illustrated here could therefore be relevant for developing a more targeted treatment plan. Importantly, the prognostic value of the clusters should be evaluated once 5-year follow-up is available.

## Conclusions

We have here identified three metabolic clusters of breast cancer, also characterized with differences at the proteomic and transcriptomic level. The metabolic clusters are not reflecting the intrinsic genetic subtypes and may give important additional information for understanding breast cancer heterogeneity. Gene enrichment analysis revealed diverse ECM characteristics among these clusters in accordance with RPPA-subtyping. The approach of combining information from several -omics levels in the same tumor shows promise in improving the understanding of breast cancer heterogeneity potentially leading to more patient specific treatment.

## Abbreviations

Ala, alanine; Cr, creatine; DCIS, ductal carcinoma in situ; ECM, extracellular matrix; ER, estrogen receptor; FDR, false discovery rate; Gly, glycine; GPC, glycerophosphocholine; GSEA, gene set enrichment analysis; HCA, hierarchical cluster analysis; HR MAS MRS, high resolution magic angle spinning magnetic resonance spectroscopy; Lac, lactate; Mc, metabolic cluster; MRS, magnetic resonance spectroscopy; MSigDB, molecular signatures database; PAM50, prediction analysis of microarrays 50; PCho, phosphocholine; PET, positron emission tomography; PLS-DA, partial least square discriminant analysis; PtdCho, phosphatidylcholine; RPPA, reverse phase protein array; SAM, significance analysis of microarrays; Tau, taurine; TCA, tricarboxylic acid; β-Glc, β-glucose

## Additional files

Additional file 1: Additional methods and references. Information of additional methods. (DOCX 22 kb) Additional file 2: Table S1. Antibodies used for reverse phase protein array (RPPA). **Table S2.** Metabolite integrals for each of the samples included in the study. **Table S3.** Significantly different expressed genes between the three metabolic clusters. **Table S4.** Significantly different expressed genes between the metabolic clusters Mc1 and Mc2. **Table S5.** Significantly different expressed genes between the metabolic clusters Mc1 and Mc3. **Table 6.** Statistically over-represented annotation terms, according to DAVID, of differently expressed genes between metabolic cluster Mc1 and Mc2. **Table S7.** Statistically over-represented annotation terms, according to DAVID, of differently expressed genes between metabolic cluster Mc1 and Mc3. **Table S8.** Gene set enrichment analysis (GSEA) result for gene ontology (GO) gene sets. Metabolic cluster Mc1 was compared with Mc2 and Mc3. **Table S9.** Gene set enrichment analysis (GSEA) result for gene ontology (GO) gene sets. Metabolic cluster Mc1 was compared with Mc2. **Table S10.** Gene set enrichment analysis (GSEA) result for gene ontology (GO) gene sets. Metabolic cluster Mc3 was compared with Mc1 and Mc2. **Table S11.** Integrated pathway analysis result from the comparison of the metabolic clusters Mc1 compared to Mc2. **Table S12.** Integrated pathway analysis result from the comparison of the metabolic clusters Mc1 compared to Mc3. (XLSX 337 kb)
