# Supplementary material for: Metabolic clusters of breast cancer in relation to gene- and protein expression subtypes
Source: Cancer Metab. 2016 Jun 27;4:12. doi: 10.1186/s40170-016-0152-x (PMC4922058; doi:10.1186/s40170-016-0152-x)
Supplement: Additional file 1: — Additional methods and references. Information of additional methods. (DOCX 22 kb) [file 40170_2016_152_MOESM1_ESM.docx]

# Additional Information:

**Additional Methods**

**HR MAS MRS acquisition and data processing.** Before HR MAS MRS experiments, 3 µL cold sodium formate in D2O (24.29mM) was added to a leak-proof disposable 30µL insert (Bruker, Biospin GmbH, Germany) as a chemical shift reference. Tissue samples were cut to fit the insert on a dedicated work station designed to keep the samples frozen [[1](#_ENREF_1)]. The insert containing the frozen sample was placed in a 4-mm diameter zirconium rotor (Bruker, Biospin GmbH, Germany) and kept at -20 ˚C for maximum 8 hours before the experiments. Samples were spun at 5000 Hz and experiments run at 5 ˚C. The samples were allowed 5 minutes temperature acclimatization before shimming and spectral acquisition. Spin-echo spectra were recorded using a Carr-Purcell-Meiboom-Gill (cpmg) pulse sequence (cpmgpr1d; Bruker) with 4s water suppression prior to a 90° excitation pulse. T2 filtering was obtained using a delay of 0.6 ms between each 180˚ pulse to suppress macromolecules and lipid signals and enhance signal from small molecules. This resulted in an effective TE of 77 ms. A total of 256 scans over a spectral region of 12 kHz was collected into 72k complex data points with an acquisition time of 3.07 s. The FIDs were multiplied by a 0.30 Hz exponential weighting function and Fourier transformed into 64k real points. Phase correction was performed automatically for each spectrum using TopSpin 3.1 (Bruker). Further preprocessing of the HR MAS spectra were performed in Matlab R2013b (The Mathworks, Inc., USA). Chemical shifts were referenced to the creatine peak at 3.92 ppm. Baseline correction was performed using asymmetric least squares [[2](#_ENREF_2)] with parameters 𝜆 = 1e7 and p = 0.0001, and baseline offset was adjusted by setting the minimum value of each spectrum to zero by subtracting the lowest value. Peak alignment was performed using icoshift [[3](#_ENREF_3)].

**Reverse Phase Protein Array (RPPA).** Tumor tissue was lysed by homogenization in lysis buffer containing proteinase inhibitors and phosphatase inhibitors. The tumor lysates were diluted in 1.33 mg/ml concentration as assessed by bicinchonic acid assay (BCA) and boiled in 1% SDS and 2-mercaptoethanol. Supernatants were manually diluted in five serial 2-fold dilutions with lysis buffer. The samples were spotted onto and immobilized on nitrocellulose-coated FAST slides. The slides were probed with 151 primary antibodies (Supplementary Table 1) in appropriate dilutions. The signal intensity was captured by a biotin conjugated secondary antibody and was amplified by Dako Cytomation-catalysed system (Dako, Glostrup, Denmark). Slides were scanned, analyzed and quantitated using MicroVigene software (VigeneTech Inc., Carlise, MA, USA) to generate spot signal intensities. These were then processed by the R package SuperCurve /version 1.01. The protein concentrations were derived from the supercurve for each sample by curve fitting, log2-transformed, and the relative concentrations were normalized by median centering of the samples for each of the antibodies [[4](#_ENREF_4)].

**Statistical analysis.** PLS-DA was performed on mean centered spectra using double cross validation [[5](#_ENREF_5)]. In the outer loop, the model was built on randomly chosen training samples (80 % of the spectra) and used to predict the class of the remaining independent test samples (20 % of the spectra). The outer loop was repeated 20 times in total. For each outer loop, 20 repetition of the inner loop was performed. Here, the training set was again divided into an inner set of training (80 % of the samples) and test (20 % of the samples) samples. The average classification result is calculated based on the outer loop, while the inner loop was used to determine the optimal number of LVs. To validate that the result is not achieved simply by random predictions, permutation testing was performed. Here Y-data (class labels for the samples) are permutated to resemble random classification. For each permutation 20 random training and test sets are chosen as described for the PLS-DA model. This was repeated 1000 times before the error distribution was compared with the classification error for the original data. P values ≤ 0.01 were considered significant. PCA and PLS-DA were performed in Matlab using PLS_Toolbox 7.5.2 (Eigenvector Research, Inc., Wenatchee, USA).

**Additional References**

1. Giskeødegård, G.F., M.D. Cao, and T.F. Bathen, *High-Resolution Magic-Angle-Spinning NMR Spectroscopy of Intact Tissue*, in *Metabonomics: Methods and Protocols*, J.T. Bjerrum, Editor 2015, Springer New York. p. 37-50.

2. Eilers, P.H., *Parametric time warping.* Anal Chem, 2004. **76**(2): p. 404-411.

3. Savorani, F., G. Tomasi, and S.B. Engelsen, *icoshift: A versatile tool for the rapid alignment of 1D NMR spectra.* J Magn Reson, 2010. **202**(2): p. 190-202.

4. Aure, M.R., et al., *Integrated analysis reveals microRNA networks coordinately expressed with key proteins in breast cancer.* Genome Med, 2015. **7**(1).

5. Smilde, A.K., et al., *Data processing in metabolomics*, in *Metabolomics in practice: successful strategies to generate and analyze metabolic data*, M. Lämmerhofer and W. Weckwerth, Editors. 2013, John Wiley & Sons.
